# Prevalence, symptomatology, risk factors and healthcare services utilization regarding paternal depression in Germany: study protocol of a controlled cross-sectional epidemiological study

**DOI:** 10.1186/s12888-019-2280-7

**Published:** 2019-09-18

**Authors:** Julia Albicker, Lars P. Hölzel, Jürgen Bengel, Katharina Domschke, Levente Kriston, Miriam A. Schiele, Fabian Frank

**Affiliations:** 1 Department of Psychiatry and Psychotherapy, Medical Centre – University of Freiburg, Faculty of Medicine, University of Freiburg, Hauptstraße 5, D-79104 Freiburg, Germany; 2 Parkklinik Wiesbaden Schlangenbad, Rheingauer Straße 47, D-65388 Schlangenbad, Germany; 3 Department of Psychology, University of Freiburg, Engelbergerstraße 41, D-79085 Freiburg, Germany; 4 Department of Medical Psychology, University Medical Center Hamburg-Eppendorf, Martinistraße 52, D-20246 Hamburg, Germany; 5 Department of Social Work, Protestant University of Applied Sciences Freiburg, Bugginger Straße 38, D-79114 Freiburg, Germany

**Keywords:** Depression, Depressive symptoms, Postpartum, Father, Men, Epidemiological study, Cross-sectional study

## Abstract

**Background:**

While postpartum depression is a well-researched disorder in mothers, there is growing evidence indicating that some fathers also develop depressive symptoms (paternal postpartum depression, PPD). A recent meta-analysis revealed a total prevalence of paternal depression during pregnancy and up to one year postpartum of 8.4%, with significant heterogeneity observed among prevalence rates. International studies suggest that PPD is characterized by additional symptoms compared to maternal postpartum depression. Furthermore, various risk factors of PPD have been identified. However, the prevalence, symptomatology, risk factors and healthcare situation of fathers affected by PPD in Germany are unknown.

**Methods/design:**

This study comprises a controlled, cross-sectional epidemiological survey administered via postal questionnaires. The primary objective is to compare the prevalence of depressive symptoms in fathers with a 0–12-month-old infant to the prevalence of depressive symptoms in men without recent paternity. Two structurally differing regions (concerning birthrate, employment status, socioeconomic structure, and nationality of inhabitants) will be included. A random sample of 4600 fathers (2300 in each region) in the postpartum period and 4600 men without recent paternity matched by age, nationality and marital status will be assessed regarding depressive symptoms using the PHQ-9. Contact data will be extracted from residents’ registration offices. As secondary objectives, the study aims to provide insights into symptoms and risk factors of PPD in fathers and to assess the current healthcare situation of fathers with PPD in Germany. In an add-on study, genetic and epigenetic mechanisms of PPD will be explored.

**Discussion:**

This study will conduct the first direct comparison between fathers in the postpartum period of one year after childbirth and a matched sample of men without a newborn child. Besides closing this research gap, the findings will provide prevalence estimates as well as insights into specific symptomatology, risk factors, and the current healthcare situation regarding fathers with PPD in Germany. The results will identify low-threshold approaches as a relevant issue for healthcare. Moreover, the findings should inform the development of PPD-specific screening instruments and healthcare offers addressing fathers with PPD.

**Trial registration:**

German Clinical Trials Register (DRKS): DRKS00013339; Trial registration date: August 20, 2018; Universal Trial Number (UTN): U1111–1218-8185.

## Background

While postpartum depression in mothers is a well-researched and recognized mental illness [1], growing evidence suggests that fathers in the postpartum period are also at increased risk of developing depressive symptoms [2–6]. Studies have indicated that the prevalence of depressive symptoms during pregnancy and the postpartum period in fathers is similar to that of mothers [4, 5, 7, 8]. A recent meta-analysis by Cameron et al. (2016) revealed a total rate of depression of 8.4% in fathers during pregnancy and up to one year postpartum, with a higher prevalence of 13% in the 3- to 6-month postpartum period [9]. An earlier meta-analysis by Paulson and Bazemore (2010) yielded a prevalence of 10.4% for depression in fathers during the postpartum period [10]. The authors found that prevalence estimates were moderated by the time of measurement, with higher depression rates of 25.6% during the 3- to 6-month postpartum period [10]. However, due to factors such as study location and methods, prevalence rates vary broadly, ranging from 4 to 25% [4, 10–13]. The varying rates of paternal postpartum depression (PPD) in different countries might be influenced by cultural biases, e.g., differing interpretations of depressive symptoms, social acceptance of mental health problems or divergent expectations with respect to paternal infant care responsibilities. Additionally, methodological aspects such as the use of different diagnostic approaches, biased translations of instruments, or differing sampling methods may also impact PPD prevalence data.

For Germany, there is currently only one longitudinal study by Gawlik et al. (2014), which examined paternal depressive symptoms over the second and third trimester of pregnancy up to 6 weeks postpartum using the Edinburgh Postnatal Depression Scale [14, 15]. The study found that 7.8% of fathers showed depressive symptoms in the postpartum period. Although these findings suggest a scientific and public importance of PPD for Germany, the study only provided information about PPD up to 6 weeks after birth, thus omitting the majority of the postpartum period. Furthermore, the available research is unable to answer the question of whether depressive symptoms are more common in fathers in the postpartum period than in men without a newborn child.

It might be assumed that PPD is characterized by male-specific symptoms [15–17]. Typical symptoms of the so-called “masked men’s depression”, for example, include rage, irritability, emotional rigidity, sleep disorders, and alcohol abuse [18–20]. In the case of PPD, first results point to additional symptoms such as feelings of inadequacy and sadness over “the loss of the old role”, irritability, indecision, impulsivity, violent behavior, avoidance behavior, and substance abuse [21–24]. However, all existing screening and diagnostic instruments for postpartum depression were developed for mothers. As these instruments only cover female-specific symptoms of postpartum depression [25], it is essential to gain distinct insights into the symptoms of PPD.

A starting point to ensure adequate care for fathers is to identify risk factors influencing the emergence and maintenance of PPD. A review by Wee et al. (2006) revealed that PPD is correlated with depressive symptoms in the partner, low relationship quality, and lack of social support [6]. A further study found that a history of severe depression, high prenatal symptom scores for depression, and anxiety were the strongest predictors of paternal depression in the postpartum period [26]. Furthermore, a high rate of comorbid depression and anxiety during the postpartum period has been found in both women and men [27]. According to Bandura [28], self-efficacy plays an important role in regulation of emotional states. High beliefs of self-efficacy make people likely to interpret potential threats as manageable challenges and help them feel less stressed in such situations [29]. In addition, several studies found a relationship between limited mental health and low general self-efficacy [30–34]. Studies also showed that high general self-efficacy beliefs were related to lower levels of depression [35, 36]. This suggests that a lack of general self-efficacy increases the likelihood of developing PPD. Other factors that have been associated with PPD are fears about birth and the father’s role, premature birth, stressful living conditions, previous depressive episodes, impaired sleep, lower socioeconomic status, and influences of cultural and gender roles [3, 10, 23, 37].

A limitation of all existing studies is that findings on prevalence, symptomatology, and risk factors of PPD focus solely on fathers in the first year after childbirth in comparison to historical control data on depressive symptoms. So far, no study has directly compared fathers in the postpartum period of one year after childbirth to a matched sample of men without a newborn child. A simultaneous investigation of depressive symptoms using the same instruments ensures the direct comparability of prevalence estimates between fathers of an infant and men without recent paternity. It provides the benefit of counteracting the risk of over- or underestimation of the relative frequency of depressive symptoms after childbirth, and thus safeguards the scientific quality of the findings. To determine whether having a newborn increases fathers’ likelihood of developing depressive symptoms (compared to not having a newborn), a valid study including a control group is necessary.

The described findings suggest that PPD is a clinically relevant problem for fathers, their families, and the healthcare system, which might currently be under-diagnosed and under-treated [24]. Both in Germany and internationally, men make less use of health care services than women [38, 39]. Traditional concepts of masculinity, feelings of shame, and the issue of stigmatization regarding PPD might prevent men from utilizing adequate treatment offers [40]. Moreover, educational materials, screening procedures, and interventions mostly focus on postpartum depression in mothers, while hardly any information exists for PPD. An increased awareness and knowledge of PPD could facilitate its identification and promote an early and adequate treatment [41]. Dennis and Chung-Lee (2006) found that both informational material and direct conversations promote the utilization of healthcare services among mothers with postpartum depressive symptoms [42]. Providing information on prevalence, symptomatology, and risk factors of PPD might reduce the stigmatization of men dealing with this issue. Therefore, it is important to capture information about the utilization of medical services of fathers suffering from PPD, about access routes in healthcare, and any available treatment offers for PPD. A detailed overview of the current medical care situation for fathers with PPD is necessary in order to offer low-threshold services and foster the implementation of preventive and curative treatment.

In sum, current international research lacks an exhaustive overview of PPD, as it failed to compare the prevalence rate, symptomatology, and risk factors of fathers within one year after childbirth with a matched control group using the same instruments. For Germany, there is no research investigating PPD throughout the entire period of one year after childbirth. Taking into account the international research gaps, this study also closes existing gaps in prevalence, symptomatology and risk factors with a particular focus on Germany. Additionally, knowledge about the healthcare situation of affected fathers can lead to the identification of low-threshold service access in the healthcare system.

## Objectives

The aim of our study is to examine the prevalence of PPD in fathers within the first year after childbirth compared to the prevalence of depressive symptoms in a matched sample of men without recent paternity. We hypothesize that the prevalence of PPD in fathers within one year after childbirth is higher than the prevalence of depressive symptoms in a matched sample of men without recent childbirth. As one of the secondary objectives, we take a closer look at the group of fathers within one year after childbirth to assess differences in PPD prevalence in different time periods. Other secondary objectives are to identify specific symptoms and risk factors of PPD. Furthermore, service utilization regarding PPD is considered as a secondary objective on a healthcare level. In an exploratory add-on study, genetic and epigenetic mechanisms of PPD will be explored.

## Methods/design

### Study design

A controlled, cross-sectional epidemiological survey in men with new paternity within one year prior to this survey (group 1: fathers of an infant) and a matched sample of men without a newborn child within one year prior to this survey (group 2: control group) will be conducted via postal questionnaires. The aim is to compare the prevalence of PPD in fathers within the first year after childbirth to the prevalence of depressive symptoms in a matched sample of men without a newborn child. As study regions, we chose two cities in Southwestern Germany with varying populations regarding birthrate, employment status, socioeconomic structure, and nationality of inhabitants: Freiburg and Mannheim, with 228,000 and 320,000 inhabitants, respectively. As social factors may influence the development of PPD in fathers and of depressive symptoms in men without new paternity, the use of two structurally different cities as study regions allows the generalizability of the results to be investigated.

As primary measure, the Patient Health Questionnaire (PHQ-9) will be used (for detailed descriptions of all instruments used in this study, see below and cf. Table 1). The PHQ-9 is a gender-independent instrument with good psychometric properties [43]. Instead of comparing the results regarding the prevalence of PPD in fathers within one year after childbirth to findings from other studies (historical controls), the data in our study will be compared to a matched sample of men without new paternity one year prior to the survey. This design allows comparative statements to be made, in which the comparison is estimated to be as unbiased as possible, thus increasing the scientific quality of the statements. Furthermore, the date of childbirth and the date of answering the questionnaire will be recorded. From these data, differences in the prevalence rates can be calculated for different time periods after the birth of the child. To assess male-specific symptoms in PPD, the survey will also include the Gotland Male Depression Scale [18]. Moreover, potential risk factors will be considered using various instruments, including the Normative Gender Role Orientation Questionnaire [44], the General Self-Efficacy Scale, and the Social Support Questionnaire [45], as well as self-constructed items (including, e.g., the child’s and mother’s perceived health status). Other potential risk factors include the presence of an earlier depressive episode, the presence of anxiety symptoms, circumstances of pregnancy and childbirth, childcare, as well as demographic factors such as age, marital status, and immigration background. The survey will also cover the healthcare situation and the utilization of obstetric services by fathers with PPD. To record healthcare utilization, the fathers will indicate which services they have used in general and with regard to mental health problems in particular during gestation and since the baby was born (based on different scales, see below and cf. Table 1). Based on the prevalence of PPD and information about healthcare utilization, it will be possible to calculate the rate of fathers who would use specific treatment. The healthcare providers which are commonly contacted during pregnancy and after childbirth (e.g., general practitioner, midwife as part of the aftercare, birth preparation courses) can be used to identify low-threshold service access to adequate healthcare and possibly show important starting points for the implementation of preventive measures or for early interventions in fathers with PPD who are currently under-served.

**Table 1:** Objectives and measurements for each group

Objective	Instrument or self-constructed item	No. of items	Group
*Primary objective*			
Depressive symptoms	Patient Health Questionnaire (PHQ-9)	9	both
*Secondary objectives*			
Prevalence differences in different time periods	Date of birth of the child, date on which the questionnaire was filled out	2	fathers of an infant
Specific depressive symptoms	Gotland Male Depression Scale	13	both
Course of depression	regarding the presence and duration of the current episode and previous episodes	5	both
Symptoms of separation anxiety	Adult Separation Anxiety Questionnaire (ASA-27)	27	both
Individuals’ tendency to interpret anxiety-associated physical sensations as threatening	Anxiety Sensitivity Index (ASI)	16	both
Participants’ subjective judgment on their own health status	“How would you describe your health in general?”	1	both
Participants’ subjective judgment on the child’s and the mother’s health status	“How would you describe the health of your infant in general?” “How would you describe the health of the child’s mother in general?”	2	fathers of an infant
Diagnosis of maternal postpartum depression	“Has a doctor or psychotherapist diagnosed postpartum depression in the child’s mother?”	1	fathers of an infant
Extent of perceived self-efficacy	General Self-Efficacy scale (GSE)	10	both
Normative Gender Role Orientation	Questionnaire of Normative Gender Role Attitudes (NGRO)	29	both
Social support	Social Support Questionnaire (F-SozU-K-14)	14	both
Use of support systems	Are you supported by other people or support systems in the care of the newborn?	1	fathers of an infant
Pregnancy and birth	child’s biological father or not; pregnancy was planned, unplanned or unwanted: whether artificial insemination was used or not; complication during pregnancy or birth; premature, miscarriage, or involved multiple births; caesarean section delivery or natural birth; having other children	10	fathers of an infant
Involvement of the father	living in the same household as the infant during pregnancy and after birth; being a single parent; present at the birth; being involved in birth preparation; nighttime care; sleep quality because of the infant; daily time with the infant; use of paternity leave	10	fathers of an infant
Sleep quality in general	“How would you rate your sleep quality in general?”	1	both
Use of medical services	based on scales of the health questionnaire of the study on Adult Health in Germany (DEGS), the German version of the Client Sociodemographic and Service Receipt Inventory (CSSRI-D) and the questionnaire for the collection of health-related resource use in the elderly population (FIMA)	2	both
Use of medical services	the use of health-related offers in the obstetric setting and the use of child-related health offers	2	fathers of an infant
Socio-demographic data	age, marital / relationship status, minimum indicator set for recording migration status, educational level, income status, professional situation	11	both

### Inclusion and exclusion criteria

The study population consists of men from the cities of Freiburg and Mannheim with new paternity within one year prior to this survey (group 1: fathers of an infant) as well as men from the same regions without a newborn child within one year prior to this survey (group 2: control group). The two groups will be matched by age, nationality and marital status. To ensure a high external validity of our results, no exclusion criteria have been established, with the exception of insufficient German language proficiency due to the nature of the survey.

### Recruitment

Contact details of potential study participants for both groups will be obtained from the residents’ registration offices of the chosen study regions of Freiburg and Mannheim, which will enable a random sample of fathers of an infant to be drawn. Due to the random selection of fathers of an infant, a balanced distribution of the children’s age between 0 and 12 months can be expected. Since a request for a sample from the residents’ registration offices takes two days to process, it is guaranteed that all included fathers of an infant will have become a father of a newborn within 12 months prior to this survey. At the same time, the residents’ registration offices of Freiburg and Mannheim will draw the matched control group. The information provided by the residents’ registration offices will include contact details as well as descriptions of the men in the samples according to the variables age, nationality and marital status. After obtaining these sample descriptions, information about the study and a consent form as well as a questionnaire will be sent by post to both groups. The questionnaire is reduced to central variables and a prepaid return envelope will be attached, as these measures are associated with an increased response rate [46].

### Primary objective

The primary objective is to assess the prevalence of PPD in fathers within the first year after childbirth (group 1: fathers of an infant) compared to the prevalence of depressive symptoms in a matched sample without recent paternity (group 2: control group). This will enable the relative frequency of depressive symptoms after childbirth to be calculated. For prevalence estimates regarding PPD in fathers of an infant and regarding depressive symptoms in the control group, respectively, the Patient Health Questionnaire (PHQ-9) will be used as a measurement instrument in both groups. With sensitivity and specificity both lying at 88% [41], the PHQ-9 shows good psychometric properties and is therefore recommended by the DSM-5 for measuring symptom severity [47]. A particular advantage of the PHQ-9 is that it can be evaluated categorically, taking into account the presence of major and additional symptoms in the diagnosis. Moreover, standard data from other studies are available for the PHQ-9 [48]. Table 1 shows the measurements to be applied to examine primary and secondary objectives in both groups (cf. Table 1).

### Secondary objectives

Secondary objectives will be addressed regarding the prevalence of PPD in different periods after childbirth, male-specific symptomatology, and risk factors of PPD. On a healthcare level, service utilization regarding PPD in fathers is considered as a secondary objective.

#### Prevalence

The prevalence in different time periods after childbirth will be measured as a secondary objective, assessed using the PHQ-9. The age of the child in weeks will be recorded by calculating the time between the date of childbirth and the date on which the questionnaire is completed.

#### Symptomatology

The identification of specific depressive symptoms will be considered as a secondary objective. The presence of male-specific depressive symptoms will be recorded using the Gotland Scale for Male Depression [18, 49], which is the only screening instrument currently available for detecting masked male depression. A validation study by Zierau et al. (2002) found an internal consistency of Cronbachs α = 0.86 for the overall scale, 0.75 for the depression subscale and 0.78 for the stress subscale. With regard to convergent validity, high correlations were observed with the conventionally used Major Depression Inventory (Spearman’s ρ = 0.77) and the WHO-5 (ρ = − 0.69) [18].

#### Risk factors

To identify risk factors of PPD, various secondary objectives will be evaluated. The course of disease was chosen as a secondary objective, as numerous studies have demonstrated its moderating influence on the development of depressive symptoms. The course of disease is not recorded in the PHQ-9 and will therefore be assessed using additional items regarding the duration of the current episode and previous episodes.

To detect present anxiety symptoms, the Adult Separation Anxiety Questionnaire (ASA-27, [50]) and the Anxiety Sensitivity Index (ASI, [51]) will be administered. The ASA-27 assesses symptoms of separation anxiety in adulthood. Principal components analysis of the ASA-27 revealed a coherent construct of separation anxiety with high internal consistency (Cronbach’s alpha = .95) and high retest reliability (r = .86; *P* < .001) [50]. The ASI questionnaire measures individuals’ tendency to interpret anxiety-associated physical sensations as threatening. It has an internal consistency of .88 (Cronbach’s alpha) and .85 (Guttman split-half reliability). Furthermore, the ASI is factorially independent of other anxiety measures [52].

Global assessments of the subjective judgment of the child’s and of the mother’s health status as possible influencing variables will be recorded using self-constructed items. Additionally, it will be asked whether a diagnosis of maternal postpartum depression is present.

The normative gender role orientation of the study participants will be evaluated using the Questionnaire of Normative Gender Role Attitudes (NGRO [44];). The NGRO focuses on internalized, personal gender role models and locates respondents between the poles of traditional vs. egalitarian standard expression. The internal consistency of the 29 items version of the NGRO is high (Cronbach’s alpha = .91). The calculation of the retest reliability gave a coefficient of r = .76 [44].

Social support will be measured using the standardized German short form of the Social Support Questionnaire (F-SozU-K-14 [45];), a unidimensional scale assessing support as perceived or anticipated support, with a focus on emotional and practical support and social integration. Additionally, the use of support systems (e.g. support from grandparents, infant care facilities, etc.) will be examined using self-constructed items.

Concerning pregnancy and birth, it is relevant to record whether the pregnancy was planned, unplanned or unwanted, whether artificial insemination was used and whether the birth was premature or involved multiple births. Furthermore, perinatal variables such as caesarean section delivery will be collected. The father should also indicate whether or not he is the child’s biological father.

The involvement of the father in the child’s care will be assessed using self-constructed items. These items cover, for example, the nighttime care, the inclusion and effectiveness of the father in the child’s upbringing and the use of paternity leave. The extent of perceived self-efficacy will be assessed using the General Self-Efficacy Scale (GSE, [53]), which covers expectations of competences e.g. a person’s subjective belief that he/she will be able to successfully deal with critical events.

The socio-demographic data collection will be based on the recommendations for epidemiological studies [54] as well as the study on Adult Health in Germany (DEGS [55];) with the objective of capturing risk factors (age, marital status, etc.). The migration background will be recorded according to the minimum indicator set for recording migration status [56]. In addition, the living conditions of young families will be assessed, including relationship status, childcare facilities, housing situation, and number and age of children.

#### Healthcare utilization

The utilization of medical services will be assessed based on scales of the health questionnaire of the study on Adult Health in Germany (DEGS [55]), the German version of the Client Sociodemographic and Service Receipt Inventory (CSSRI-D [57, 58];) and the questionnaire for the collection of health-related resource use in the elderly population (FIMA [59];). In addition, the use of health-related offers in the obstetric setting and the use of child-related health offers will be recorded.

### Statistical methods

#### Dealing with dropouts (unit missing)

Based on the data of the residents’ registration offices, it will be possible to describe differences in the composition of the population and the respondents of the two samples (fathers of an infant and control group) in terms of marital status, nationality, and age. The influence of these covariates on the response behavior can be controlled using logistic regressions. If there are significant differences between the population and the respondents of the two samples, a statistical adjustment of these differences will be made in the evaluation.

#### Dealing with missing values (item missing)

In psychometrically tested instruments, missing values are handled according to the instructions in the respective manual. If there are no recommendations, up to 30% of the missing data is replaced by the expectation-maximization algorithm method [60].

#### Analyses

Relative frequencies will be calculated for nominal-scaled variables. For ordinal-scaled and non-normally distributed interval-scaled measurements, the median is used as the preferred measure of central tendency. For interval-scaled variables, the arithmetic mean and standard deviations will be calculated. The choice of the respective statistical method for group comparisons depends on the scale level of the dependent variable. Although a matched sampling will be performed, this information cannot be used in the evaluation due to the anonymity of the survey. The evaluation therefore requires the use of independent samples techniques. Nominal distributions will be compared using Chi-square and Fisher’s exact tests. Parametric tests (t-test, analysis of variance) will be used for interval-scaled measurements. The effect of socio-demographic and other influencing factors will be calculated using logistic regressions.

#### Sample size calculation

The study will investigate whether a significant difference can be observed between the prevalence of PPD in fathers within the first year after childbirth and the prevalence of depressive symptoms in a matched sample of men without recent paternity. The a priori calculation of statistical power is based on a point prevalence of 5.3% in the control group (95% confidence interval 4.3–6.3%), which corresponds to the incidence of depressive symptoms in men according to the PHQ-9 standard data [48]. Compared to reported prevalence rates, an increase of more than 50% in the point prevalence in fathers of an infant would be relevant. This corresponds to a point prevalence of at least 8.0%. According to the meta-analysis by Cameron et al. (2016), the point prevalence of PPD in fathers in the first year after childbirth lies between 7.2 and 9.6% [9]. Therefore, a potential prevalence of 8.0% is not only a realistic outcome considering the results of previous studies, but would also represent a relevant increase in the rate of depressive symptoms. To be able to detect the difference between the population prevalence of 8.0% in fathers of an infant and 5.0% in the control group with a significance level of 0.05 and a power of 80% in a Chi-square test, data from a total sample size of 2300 participants are necessary.

In Freiburg and Mannheim together, about 5500 children are born each year [61, 62]. Out of these, 4600 fathers of an infant will be randomly selected, meaning 2300 fathers of an infant per city. In addition, a control group (*n* = 4600; 2300 per city) matched according to age, nationality, and marital status will be generated. Thus, a total of 9200 potential participants will be contacted. Based on response rates from other anonymized postal surveys, a dropout rate of about 75% is expected for this study. Already included in these dropout calculations are those men who cannot participate due to incorrect address information. However, the obtained sample size will be sufficiently large to reveal small differences in the prevalence of depressive symptoms among the fathers of an infant (ω = 0.1) for each month after birth with a power > 80% using the Chi-square test.

### Trial status

Enrolment for the trial began in September 2018. Recruitment and data collection continued until March 2019. As of August 2019, data management and data analysis is ongoing.

### Exploratory genetic add-on study

First results indicate the relevance of genetic and epigenetic factors in PPD, which show a complex interaction with psychosocial and environmental aspects [63, 64]. Epigenetic mechanisms include, for example, the methylation of the cytosine-pyrimidine ring in CpG-dinucleotides of DNA, which is associated in most cases with a repression (“silencing”) of the gene transcription [65, 66]. Animal and human studies have shown that epigenetic processes represent flexible and temporally dynamic mechanisms that are significantly influenced by environmental factors [67, 68]. In terms of depression in general, changes in DNA methylation patterns have been detected in classical candidate genes [69], and altered methylation patterns have also been reported in the context of postpartum depression [70, 71]. However, the current knowledge refers primarily to epigenetic markers of postpartum depression in mothers. Therefore, epigenetic factors regarding PPD in fathers will be examined in an exploratory add-on study of this project. The add-on study aims to examine currently unexplained neurobiological mechanisms and genetic and epigenetic factors, respectively, as risk markers for the development of PPD in fathers compared to the development of depressive symptoms in men without recent paternity. Genetic polymorphisms or epigenetic processes such as DNA (hydroxy) methylation patterns will be determined in candidate genes that are selected based on a priori hypotheses. The study design follows the design of the main study described above. Together with the questionnaire of the main study, potential participants will receive information and a consent form to take part in a continuing epigenetic study. For the recruitment, all fathers of an infant and all men in the control group will be asked whether they are willing to give a DNA sample by swabbing the oral mucosa for genetic and epigenetic investigations. Participants who give informed consent will be provided with a home use kit, with which they will make a smear of the oral mucosal cells, from which the DNA for genetic and epigenetic analysis will be extracted. The obtained genetic and epigenetic markers will be related to the parameters collected within the main study. Individuals with severe neurological or somatic diseases, a non-Caucasian origin, or excessive alcohol or drug consumption will be excluded. Regarding the analyses, DNA extraction from oral mucosal cells will be carried out at the Laboratory of Psychiatric Genetics and Epigenetics of the Department of Psychiatry and Psychotherapy of the Medical Centre, University of Freiburg and in laboratories of cooperation partners for special analyses.

## Discussion

Our controlled cross-sectional epidemiological study aims to compare the prevalence of PPD in fathers within the first year after childbirth with the prevalence of depressive symptoms in a matched sample of men without new paternity. Furthermore, our study should reveal differences regarding the prevalence of PPD in fathers in different time periods within one year after childbirth. Additionally, potential male-specific symptoms and risk factors of PPD in fathers will be investigated. A further goal is to assess the current healthcare situation of fathers with PPD in Germany. Our findings will contribute to closing the above-mentioned research gaps and offer possibilities to refine healthcare options for fathers with PPD. Compared to previous studies, our study has several methodological strengths. A central advantage is the direct comparison of the prevalence of PPD in fathers of an infant with the prevalence of depressive symptoms in a matched group of men without recent paternity from the general population. The simultaneous detection of depressive symptoms using the same measurement instrument will minimize the risk of producing an estimation bias of the relative prevalence of PPD and thus increase the external validity of the results. The large sample size compared to previous studies will allow us to perform a variety of subgroup analyses. Furthermore, the large sample size and the recording of the date of childbirth and the date of responding to our questionnaire will allow us to examine prevalence differences for several time periods within one year after childbirth and to analyze specific symptoms of PPD and risk factors. A further advantage of our study lies in the consideration of the healthcare situation of fathers with PPD in Germany – an issue that has not been considered in the research so far. By examining these aspects, low-threshold service access can be revealed, enabling important hints to be derived for the development of adequate care options for fathers with PPD. The identification of risk factors for the development of PPD in fathers will allow an early and specific treatment for these men through the utilization of adequate healthcare offers. Finally, genetic and epigenetic risk patterns of PPD to be identified in the exploratory add-on study might serve as future biomarkers for early interventions or even personalized preventive measures in men at risk of PPD.

A potential limitation lies in the method of recruitment, which could produce a selection bias in the sample and thus restrict the external validity of the results. A further limitation is that study participants have to self-administer a high number of measurement instruments. This might hinder the feasibility of the trial and reduce the response rate. We have tried to address this issue by naming a contact person from the study team in case of questions, by giving examples on how to answer the questionnaire, and by reducing the questionnaire to relevant aspects. As we will also assess the general health status and other diagnoses such as anxiety, it will be possible to detect some of the potential confounding factors.

Our study will provide insights into the issue of PPD in fathers. Currently, there is no study on PPD using a matched control group. Studies conducted to date compared their results with historical control data, which carries the risk of over- or underestimation of the relative frequency of depressive symptoms after childbirth. A simultaneous detection of depressive symptoms, of symptomatology, and of risk factors using the same instruments will enhance the comparability of the results and thus ensure the scientific quality of the concluding statements. Surveying the healthcare situation will provide opportunities for the practice-oriented design of current care and thus information on the development of screening methods and healthcare concepts. Based on the findings of our study, further studies should address the standardization of measurement methods to identify PPD as well as male-specific healthcare interventions in fathers.

## Data Availability

Not applicable.

## Abbreviations

ASA-27: Adult Separation Anxiety Questionnaire
ASI: Anxiety Sensitivity Index
c.f.: compare with
CSSRI-D: German version of the Client Sociodemographic and Service Receipt Inventory
DEGS: German Health Interview and Examination Survey for Adults
DNA: Deoxyribonucleic acid
e.g.: for example
et al.: and others
etc.: et cetera
FIMA: questionnaire for health-related resource use in the elderly population
F-SozU-K-14: Social Support Questionnaire
GSE: General Self-Efficacy scale
NGRO: Questionnaire of Normative Gender Role Attitudes
PHQ-9: Patient Health Questionnaire
PPD: Paternal postpartum depression
Tab.: Table
vs.: versus
WHO-5: World Health Organization – Five Well-Being Index
